# Brazil's battle against *Rhipicephalus (Boophilus) microplus* ticks: current strategies and future directions

**DOI:** 10.1590/S1984-29612024026

**Published:** 2024-06-21

**Authors:** Guilherme Marcondes Klafke, Patrícia Silva Golo, Caio Marcio Oliveira Monteiro, Lívio Martins Costa-Júnior, José Reck

**Affiliations:** 1 Instituto de Pesquisas Veterinárias Desidério Finamor – IPVDF, Eldorado do Sul, RS, Brasil; 2 Universidade Federal Rural do Rio de Janeiro – UFRRJ, Seropédica, RJ, Brasil; 3 Universidade Federal de Goiás – UFG, Goiânia, GO, Brasil; 4 Universidade Federal do Maranhão – UFMA, São Luís, MA, Brasil

**Keywords:** Cattle tick, integrated parasite management, resistance, synthetic acaricide, multiple resistance, Carrapato bovino, controle integrado de parasitos, resistência, carrapaticidas sintéticos, resistência múltipla

## Abstract

Ticks are parasitic arthropods that cause significant economic losses to livestock production worldwide. Although *Rhipicephalus (Boophilus) microplus*, the cattle tick, occurs throughout the Brazilian territory, there is no official program to control this tick, which is the vector of tick fever pathogens. We address the situation of *R. (B.) microplus* resistance to synthetic acaricides in Brazil, including cattle tick management; the status of tick resistance per Brazilian state; the history of resistance occurrence of different acaricides; multiple resistance occurrence; and the main strategies for integrated tick management. Tick control in Brazil is characterized by management errors. Local laboratories affiliated with federal and state research institutions and universities employ the Adult Immersion Test as a primary diagnostic method to assess acaricide resistance to topically applied drugs. Only three states (Acre, Amapá, and Amazonas) have no reports on resistant populations. Misinformation on tick control strategies, misuse of available products for tick control, no adoption of Integrated Parasite Management (IPM) practices, low technical support to producers, and the high-speed emergence of acaricide-resistant tick populations are the main problems. We also propose a list of needs and priorities for cattle tick control regarding communication, research, and policies.

## Resumo

Carrapatos são artrópodes parasitos que causam perdas econômicas significativas na produção de bovinos em todo o mundo. Embora o carrapato bovino - *Rhipicephalus (Boophilus) microplus -* esteja distribuído em todo o território brasileiro, não há um programa oficial de controle do parasito. Nesta revisão, foi abordada a situação da resistência de *R. (B.) microplus* aos carrapaticidas sintéticos no Brasil, incluindo: controle do carrapato; estado da resistência por estado; histórico da ocorrência de resistência a diferentes carrapaticidas; resistência múltipla; e as principais estratégias para o manejo integrado de carrapatos. No Brasil, laboratórios locais empregam empregam, inicialmente, o teste de imersão de adultos como método diagnóstico de resistência aos carrapaticidas. Apenas três estados (Acre, Amapá e Amazonas) não têm relatos de populações resistentes. O controle de carrapatos, no Brasil, é caracterizado por erros de manejo, há desinformação sobre estratégias de controle de carrapatos, uso inadequado dos produtos disponíveis, falta de adoção de práticas de manejo integrado de parasitos (MIP), baixo suporte técnico aos produtores e a rápida emergência de populações de carrapatos multirresistentes a carrapaticidas. Ao final, propõe-se uma lista de necessidades e prioridades para o controle de carrapatos em bovinos com relação à comunicação, pesquisa e políticas públicas.

## Introduction

Ticks are generally regarded as the ectoparasites that cause the greatest economic losses to livestock production in the world (FAO, 2022). *Rhipicephalus (Boophilus) microplus* (Canestrini, 1887), a highly invasive tick, is found in tropical and subtropical regions with cattle production in Asia, Africa, and the Americas. In Brazil, it is the most important ectoparasite of cattle and caused estimated losses exceeding three billion US dollars in 2014 (Grisi et al., 2014). Worldwide, synthetic acaricides are the main resource used in the therapeutic control of ticks in cattle. Nevertheless, the intensity and frequency of exposure to acaricides play a significant role in the selection of resistant tick populations. Acaricide resistance in ticks is a complex phenomenon influenced by a combination of genetic, environmental, and management factors. Inadequate monitoring and failure at surveillance also contribute to increased resistance to synthetic acaricides. Without adequate monitoring, it is difficult to detect the early signs of resistance, and this delayed detection can lead to its rapid spread within tick populations. Whilst innovative and eco-friendly non-synthetic chemical technologies for tick control and integrated tick management exist, their limited implementation in the field can be attributed to the absence of readily available commercial products and a lack of validation through practical operational use.

Traditionally, in Brazil, zebu breeds dominated the beef cattle sector, whereas dairy cattle were primarily composed of taurine or crossbred breeds. An exception is the State of Rio Grande do Sul, where beef cattle consistently exhibited a significant taurine genetic presence (Sousa e Silva et al., 2014). Consequently, the prevalence of tick-related issues was primarily concentrated in dairy regions characterized by taurine or crossbred cattle and in beef cattle ranches in Rio Grande do Sul. In recent times, the pursuit of early maturity and enhanced meat quality has led to a surge in industrial crossbreeding or the introduction of taurine breeds. This trend, however, has not been limited to specific regions, contributing to an increased susceptibility of beef cattle to ticks across Brazil. In the last 50 years, there has been a shift and migration of the bovine population towards the North of the country, where the tick challenge tends to be greater than in the central-southern regions (McManus et al., 2016). Cattle numbers in the Brazilian Amazon biome have increased 10-fold, whereas the numbers in the Atlantic Forest biome (East Brazil) have increased by 23.6% (IBGE, 2017).

Despite official and governmental programs for tick control or eradication in some countries (i.e., Australia, Argentina, Uruguay, Mexico, USA), there is no official program to control or eradicate *R. (B.) microplus* in Brazil. This means that all decisions on tick control, such as what acaricide to use, when to treat the animals, how many treatments to perform, when to change from one acaricide to another, completely rely on the farmers and their technical staff. For more information on tick control policies in other countries, information is available elsewhere (Northern Territory Government of Australia, 2023; SENASA, 2023; Uruguay, 1956; México, 2015; USDA-APHIS, 2023).

The only local policy regarding tick control programs in Brazil occurs in an officially recognized cattle tick-free area in the two southernmost municipalities of Brazil: Santa Vitória do Palmar and Chui, in Rio Grande do Sul State. These two municipalities are located south of the latitude 32º S, which is an ecologically unfavorable zone for the cattle tick. They are on the border of the Uruguayan province of Rocha (a cattle tick-free area) to the South and separated from the rest of Brazil in the North by the “ESEC Taim”, a wildlife refuge area. Additionally, in the west and east, this tick-free area is delimited by water bodies (Pereira et al., 2023). In this region, the transit of livestock from infested areas into the free zone has been controlled by the state veterinary service since 1951 (Rio Grande do Sul, 1951).

Other initiatives, even though still in their early stages, should be mentioned, such as the establishment of a working group aimed at conducting studies and preparing technical documents to support the formulation and implementation of state parasite control programs (including cattle tick) in the states of Maranhão (AGED/MA) and Rio Grande do Sul (SEAPI/RS). Additionally, there is the provision of logistical support and the distribution of acaricides by the municipal government of Poço das Trincheiras, State of Alagoas, to support livestock breeders. Whilst these initiatives are highly welcome, they require technical and scientific analysis, planning, and ongoing commitment of the government, veterinary services, and producers.

## Cattle Tick Control in Brazil

In Brazil, the prevailing strategy for tick control in the field heavily relies on the use of synthetic acaricides. Presently, there are approximately 250 products marketed for tick control in Brazil (Brasil, 2023a). Unfortunately, integrated parasite management (IPM) practices are often neglected, thereby imposing a substantial burden on the expected efficacy of the employed acaricides. Moreover, relying solely on synthetic acaricides for control may result in an overuse of these drugs on farms. This overreliance raises concerns about the sustainability of tick control strategies and the potential development of resistance in tick populations. To ensure a more balanced and sustainable approach, there is a growing need to promote and implement IPM practices that encompass a range of strategies beyond the sole use of synthetic acaricides.

As mentioned above, decisions on cattle tick control in Brazil are made by the farmers and, eventually, by the veterinarians working in the field. Although there is plenty of information available for the correct use of acaricides, the misuse of these products is common. One of the main examples of the improper use of these drugs is incorrect dosing, which may be associated with the erroneous dilution of acaricides, insufficient amounts of product applied over the animal, and incorrect animal weight estimation, among others (authors’ observations).

Manual pumps are widely employed to apply acaricides in Brazil. Although this approach is a low-cost strategy for farmers, it is frequently incorrectly used. According to our experience, most farmers use an insufficient volume of acaricide solution per animal. In addition, it is not infrequent to observe some farmers spraying just some parts of the animal’s body. Also, for the correct application of the acaricides with a manual pump, adequate cattle restraint is necessary. This has a major impact considering that most farms in Brazil, especially the small ones, do not have any restraint system available, such as cattle squeeze chutes (authors’ observations).

Another point that deserves attention is the incorrect management of cattle spray races and dipping vats. In our experience, several mistakes may also occur in the use of these tools. For dipping vats, the accumulation of excessive organic material at the bottom of the vat, off-label acaricide use, insufficient homogenization of the acaricide in the vat, incorrect acaricide recharge, and water infiltration in the vat are among the major causes of treatment failure. Similarly, in spray races, major failures can arise from insufficient sprayers numbers, nozzle clogging, low water pressure, and inadequate corridor length in certain models. Additionally, it is essential to highlight the remarkably low availability of personal protective equipment (PPE) for the workforce responsible for acaricide management (authors’ observations).

One pivotal question for synthetic acaricides is: what most influences the choice of which acaricide to use on the farm? This question has been posed to numerous farmers in southern Brazil over the past few years. A significant portion of respondents, around 50%, indicated that their primary criterion for choosing an acaricide is the recommendation provided by the salesperson at the store. Interestingly, less than one-third of the farmers stated that they received support from veterinary practitioners regarding tick control. Only a small part of the farmers (less than 10%) declared that they chose the acaricide based on laboratory tests (IPVDF, unpublished data).

These features highlight that any further strategies regarding tick control must include continuous education and training programs for the farmers. Moreover, the role of veterinary practitioners as key actors in cattle tick control programs must be reinforced, providing continuous education for these professionals. Other strategies may be needed to improve the availability of technical support to the farmers, particularly those operating at small scales.

## *R. (B.) microplus* Resistance to Synthetic Acaricides

According to the concept revised by Stone (1972) and later adopted by several authors (FAO, 2004), drug resistance “is the ability of a population of arthropods to tolerate doses of drug which would prove lethal to the majority of individuals of a normal population of the same species”. This concept fundamentally involves a comparative analysis between a particular population and most of the species. However, a critical question arises: do we genuinely possess comprehensive knowledge regarding the susceptibility status of most tick populations? In a scenario of widespread resistance, we must be careful and consider the space-time variations. For instance, in Brazil, today, a substantial portion of tick populations exhibit resistance to pyrethroids, rendering the drug non-lethal to most individuals within these populations.

In 2004, the Food and Agriculture Organization of the United Nations (FAO, 2004) defined drug resistance as “the detection, by means of sensitive tests, of a significant increase in the number of individuals within a single population of a species of parasite that can tolerate doses of drug(s) that have proved to be lethal for most individuals of the same species”. This brings a novel element to the concept discussion: the need for sensitive detection methods. In general, the concepts do not specify whether the resistance determination was strictly dependent to be demonstrated *in vivo* (in naturally or artificially infested hosts) or if it would be determined *in vitro* (laboratory tests). Since the demonstration of the susceptibility of a parasite population in living models is challenging, it is rarely reported in the literature. This means that diagnostics depend upon *in vitro* tests to identify the drug susceptibility of a population, and the results can be considered a good indication of the resistance profile of a given population (Torrents et al., 2020). Although unpractical, field tests possibly provide more accurate results regarding the resistance profile of tick populations (Reck et al., 2014a; Torrents et al., 2020).

Recently, the World Health Organization (WHO, 2023) has adopted a concept that considers the variable time, as following: “resistance occurs when bacteria, viruses, fungi, and parasites change over time and no longer respond to medicines, making infections harder to treat and increasing the risk of disease spread, severe illness, and death”. Despite that, this concept relies on a comparative approach, i.e., between the population´s present status and the population´s status at the time the drug was introduced. A comparison is difficult since for most populations, information on their previous drug susceptibility was not available. However, it is worth highlighting that it would be the ideal scenario to determine the profile that is expected for a species, based on the knowledge of the dose-response effect in different populations. This is in line with the concept of resistance presented by the FAO, allowing the determination of the resistance profiles of ticks through comparing the mortality values of a given population in relation to what is expected for the species. One way to address these limitations, although it still relies on a comparative approach, is to use a susceptible reference population/strain. These reference strains are usually isolated from the field previously to drug exposure and maintained under controlled conditions without further drug exposure. For *R. (B.) microplus* ticks, two susceptible reference strains were maintained and largely used in South America, namely “Mozo” and “Porto Alegre” strains (Lovis et al., 2013; Reck et al., 2014a).

Another essential point which always needs to be included in discussions on the drug resistance concept is that it is strictly an inherited characteristic, which means that it can be transmitted to the offspring. It is essential to allow a further understanding of the selection mechanisms (FAO, 2004).

Expanding the concept and considering all points raised above, we can try to define drug resistance in ticks as a genetic phenomenon in which some individuals are selected over time, leading to an increasing ability of the population to survive in the presence of drugs designed to kill them in comparison to the majority of the individuals of the species (at least at the moment of the drug release in the market) or to a reference susceptible strain, as demonstrated by validated diagnostic tests.

Another crucial aspect to consider is the distinction between drug resistance and field efficacy. Whilst it is anticipated that a population of ticks resistant to a particular drug will exhibit reduced efficacy when exposed in the field, compared to a susceptible strain, the classification of a population as resistant does not imply complete ineffectiveness of the acaricide in the field. The diagnosis of drug resistance often relies on *in vitro* tests, and it is crucial to recognize that direct extrapolation of *in vitro* results to *in vivo* situations may not always be accurate.

## Reports on Cattle Tick Resistance

The historical massive use of chemical acaricides led to reports of multiple resistance against the main classes of acaricides in all regions in which the cattle tick is found. Of the seven classes of chemical acaricides marketed in Brazil, resistance to six of them has been reported (Reck et al., 2014a; Valsoni et al., 2021). To date, there is no report of resistance against isoxazolines, which have been released to the market for cattle in 2022.

**Figure 1:**
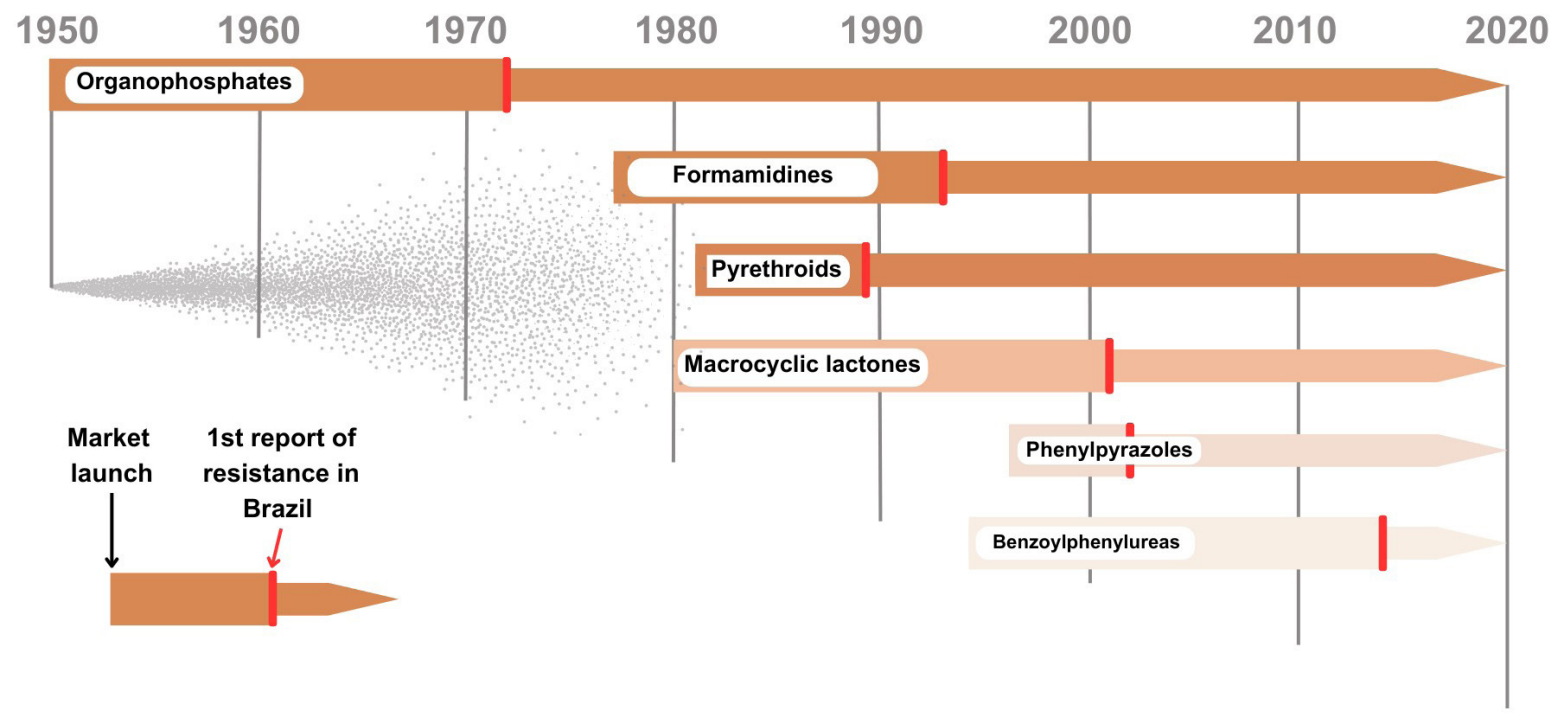
History of acaricide resistance in the cattle tick in Brazil. The data were based on indexed scientific articles or freely available conference papers. Details of first resistance case-reports in Brazil and other countries can be found on the Supplementary Table 1.

The timeline of resistance reports of chemical classes of acaricides still used today is shown in Figure 1 and Supplementary Table S1. Organophosphates (OP) were released to the market in 1950, and 14 years later, the first report of resistance was registered in Australia (Shaw & Malcolm, 1964). Twenty-two years after market release, the first report of OP resistance in Brazil was registered in Rio Grande do Sul State (Arteche, 1972). Amitraz started to be used in 1975, and the first report of resistance occurred after a short period in Australia. Sixteen years after it was registered in Brazil, in 1977, amitraz resistance was documented in the country in 1993, in Rio de Janeiro State. Synthetic pyrethroids (SP) were introduced to the market in 1981, and the first reports of resistance were simultaneously published in 1989, both in Australia (Nolan et al., 1989) and in Brazil, in Rio Grande do Sul State. Macrocyclic lactones (ML) were launched in 1980, and resistance was first registered in Brazil in 2001, in Rio Grande do Sul State (Martins & Furlong, 2001). In 2010, resistance to ML was registered for the first time in another country (Mexico) (Perez-Cogollo et al., 2010). Fipronil was released to the market in 1996, and in less than a decade, resistance was observed in Brazil, in 2004 in Minas Gerais State. A few years later, resistance to fipronil was reported in another country (Uruguay) (Cuore et al., 2007). Fluazuron has been available on the market since 1994, and the first report of resistance was published in 2014 in Brazil, in Rio Grande do Sul State (Reck et al., 2014a). In 2022, fluazuron resistance was recorded in another country (Argentina) (Torrents et al., 2022). Although mixtures of different acaricides cannot be considered chemical classes *per se*, they constitute a widely used group of drugs in Brazil. Among them, the most important mixtures in use in Brazil are those containing organophosphates + pyrethroids (OP+SP). The first OP+SP mixture was launched on the Brazilian market in 1982, and 13 years later, in 1995, resistance to OP+SP mixture was registered for the first time in Brazil, in Rio Grande do Sul State. Although OP+SP mixtures are common in Brazil, they do not seem to be widely used in other countries. Resistance to OP+SP mixtures was registered in another country for the first time in 2014 (Uruguay) (Cuore & Solari, 2014). The history presented above regarding the acaricide resistance timeline proves that independently of novel drugs and chemical classes becoming available, the use of chemical acaricides will always face the emergence of resistance cases. In this context, it is crucial to change the way tick control was performed in the last 100 years, mainly to reduce the strict dependence on the use of chemical acaricides and their massive use in the field.

## Acaricide Resistance Status in Brazil

Cattle tick populations resistant to acaricides can be diagnosed through *in vitro* laboratory tests. The most used tests are bioassays conducted on engorged female ticks and tick larvae. The Food and Agriculture Organization of the United Nations (FAO) recommends using the larval packet test (LPT) for acaricide resistance diagnosis in cattle ticks, as mentioned in their 2004 guidelines (FAO, 2004). The method was adapted from Stone & Haydock´s (Stone & Haydock, 1962) technique, that consisted in exposing the tick larvae in filter paper packets impregnated with the active ingredients dissolved in oil. Nevertheless, its application in Brazil for resistance detection is restricted to a few laboratories that mostly used it in epidemiological surveys (Mendes et al., 2011; Mendes et al., 2013; Klafke et al., 2017; Vilela et al., 2020) and in the description of acaricide resistance mechanisms in ticks (Pohl et al., 2011; de La Canal et al., 2021).

In Brazil, diagnostic service laboratories frequently use the adult immersion test (AIT) for acaricide resistance diagnosis, which was originally described by Drummond et al. (1973). The technique was modified to assess resistance by evaluating the reproductive performance of engorged females immersed in acaricidal solutions prepared at the recommended concentration for use in dipping vats or sprays. This is a simple and economical test that can be performed using readily available commercial products. Whilst this technique has some limitations, such as its restricted reproducibility and repeatability, as mentioned by Jonsson et al. (2007), it remains a widely adopted method. Also, as previously pointed out, it is possible that AIT was not always able to discriminate between resistant and susceptible populations, which may account for false-negative results in the test (Jonsson et al., 2007). A significant majority of published reports on acaricide resistance to topical acaricides such as cypermethrin, amitraz, chlorfenvinphos, and OP+PS mixtures in Brazil rely on the AIT method. The common use of AIT in Brazil is significant due to the number of products that are combinations of acaricides (e.g., cypermethrin/chlorpyriphos). To the best of our knowledge, there is no standardized LPT for the detection of resistance to these mixtures. Additionally, fluazuron cannot be tested with larval tests due to its mode of action (acarine growth disruptor). It is also important to note that most laboratories utilize AIT to provide guidance for veterinarians/producers in selecting an acaricide product.

Conversely, the larval immersion test in microtubes (m-LIT), which was proposed by Sabatini et al. (2001), is a prevalent choice for diagnosing resistance to injectable drugs such as macrocyclic lactones (Klafke et al., 2012; Singh et al., 2015; Chaparro-Gutiérrez et al., 2020; Torrents et al., 2020). The method was also adapted for fipronil and amitraz resistance detection (Castro-Janer et al., 2010; Mendes et al., 2013). The m-LIT involves exposing tick larvae to serially diluted acaricide solutions, which can be prepared using either commercial formulations or technical active ingredients dissolved in ethanol or acetone along with Triton-X 100. Following the incubation period, the mortality rate of the larvae is documented, and these data are then used to calculate the lethal concentrations. These concentrations are subsequently employed to establish resistance ratios, allowing for the differentiation between resistant and susceptible populations.

For all tests mentioned above (LPT, AIT, m-LIT), results are obtained just after six weeks. This time is needed since AIT considers the acaricide effect both at oviposition and larvae hatching, and for LPT and m-LIT it is needed to rear larvae in the laboratory from the engorged females collected from cattle. Thus, considering the life cycle of cattle ticks, which usually take two weeks for oviposition, and another three to four weeks for larvae hatching, the results can be obtained after a six-week period. Ideally, novel tests should achieve results in a shorter time to facilitate the decision on tick control programs.

Nevertheless, there is no perfect test for resistance detection. Importantly, one cannot directly translate the bioassay results to the field efficacy of the acaricide because there are intrinsic features of the laboratory assays that do not correspond to the conditions in the field. Nevertheless, the submission of ticks to the laboratory to test for acaricide resistance/susceptibility is the first step for rational tick control. The laboratory assays can provide an indication if the local population (or strain) has a presence of resistant ticks that would survive acaricidal treatments; if this is the case, acaricide replacement should be considered.

**Figure 2:**
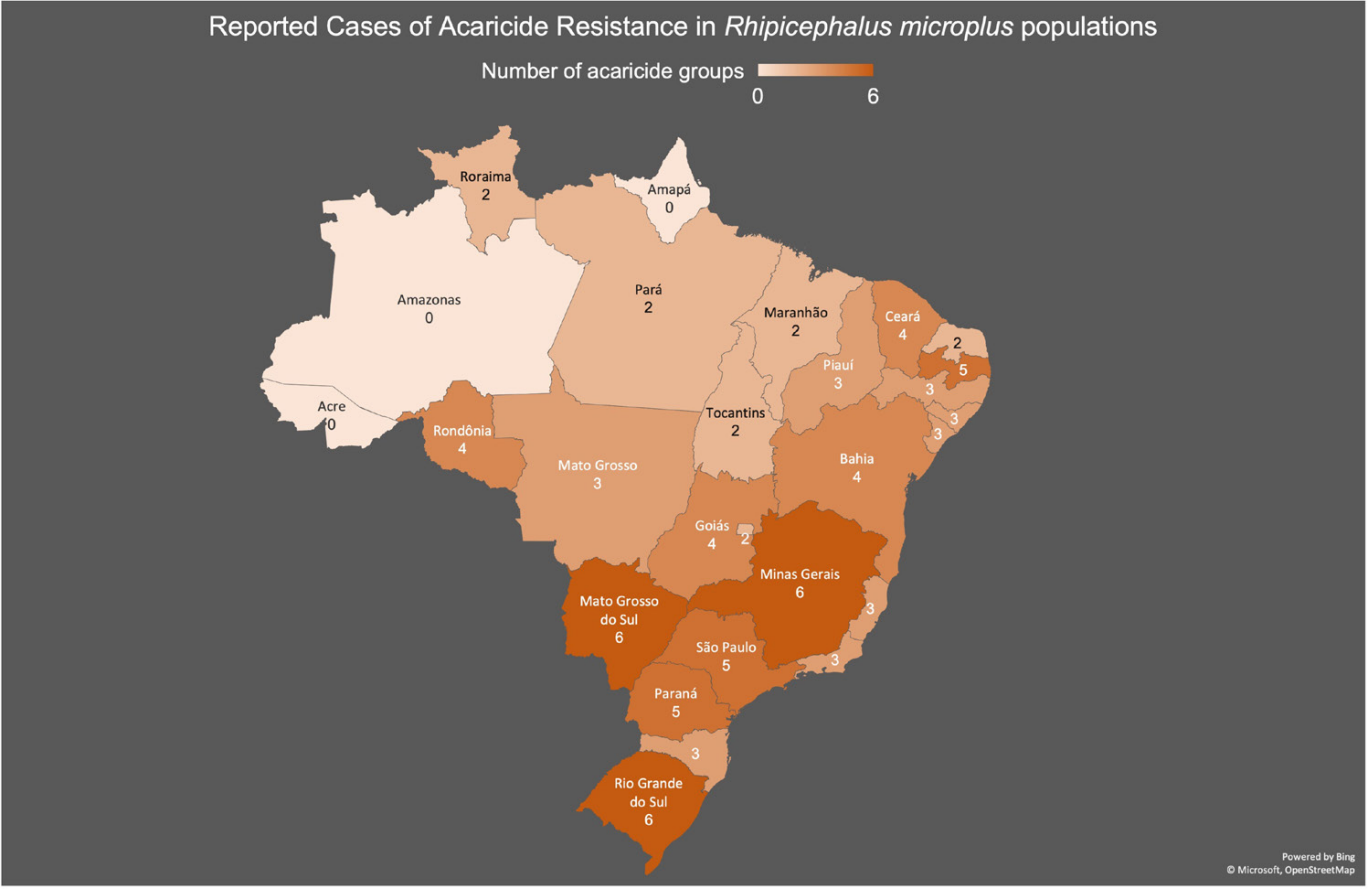
Reported cases of acaricide resistance in *Rhipicephalus microplus* in Brazil.

Through laboratory bioassays, a significant number of cases of acaricide resistance in *R. (B.) microplus* in Brazil have been documented (refer to Figure 2 and Supplementary Table 2). Notably, the states of Rio Grande do Sul (RS), Minas Gerais (MG), and Mato Grosso do Sul (MS) reported multiple instances of acaricide resistance across all chemical classes. These states, recognized as major producers of beef and dairy products in Brazil, have reference diagnostic laboratories for acaricide resistance (Instituto de Pesquisas Veterinárias Desidério Finamor - IPVDF, Embrapa Gado de Leite, and Embrapa Gado de Corte, respectively). A noticeable gap in resistance data reporting appears to exist in the Amazon region, encompassing Pará, Mato Grosso, Rondônia, Roraima, Tocantins, Maranhão, Amazonas, Acre, and Amapá. The ecological conditions in this region favor the development of *R. (B.) microplus*, particularly as cattle production gains prominence. It is therefore plausible to infer an elevated frequency of treatments per year in cattle operations, especially those involving European breeds or their crosses. However, the absence of acaricide resistance reference laboratories in the region may contribute to its underrepresentation in acaricide resistance mapping within Brazil. On a broader scale, multiple acaricide resistance (MRA) seems to be widely distributed throughout Brazil. Despite this ubiquity, the factors underlying this resistance remain undefined. It is imperative to acknowledge the potential impact of systematic errors in the application of synthetic acaricides, further emphasizing the need for comprehensive research and monitoring to better understand and address the challenges associated with acaricide resistance in *R. (B.) microplus* across Brazil.

The uniformity brought about by a network of reference laboratories would enhance the consistency and reliability of data collection and analysis. This, in turn, facilitates a more comprehensive understanding of tick-related challenges and the development of effective control strategies. By harmonizing methodologies and reporting mechanisms, the network would contribute to a more homogeneous and assertive approach to tick control.

## Drug Residues and Sustainable Tick Control

The control of parasites, including cattle ticks, is directly linked to One Health, improving animal welfare and productivity (animal health), reducing environmental impact (environmental health), and enhancing the quality of food for humans (human health). A relevant data point in Brazil is that, according to analyses conducted by the national plan for residue and contaminant control of the Ministry of Agriculture from 2020 to 2022 (Brasil, 2023a), acaricides were among the most common causes of non-compliance in both milk and beef. Out of 10,796 samples of slaughtered cattle analyzed during this period, 26 non-compliant samples were detected, with 15 (57.7%) containing acaricides (8 with fipronil, 3 with abamectin, 3 with doramectin, and 1 with ivermectin). In the case of milk samples analyzed (n = 2,452), 24 non-compliant samples were detected, with 10 (41.6%) containing acaricides (4 with ivermectin, 2 with abamectin, 2 with chlorpyrifos, 1 with doramectin, and 1 with ethion). It should be mentioned that this number may be underestimated, since it only considers cattle slaughtered under federal inspection, while millions of animals were processed each year in slaughterhouses under state and municipal inspection. These data demonstrate the importance of tick control in both animal health and public health, emphasizing the significance of managing acaricide resistance as it contributes to an increased number of acaricide treatments.

## Control of Multiple Acaricide-resistant Cattle Ticks

Usually, the scientific literature and technical information recommend “strategic tick control protocols” as resources to reduce tick burden, minimize the occurrence of control failures, and slow the selection for acaricide resistance. However, almost all protocols of strategic tick control refer to scenarios in which tick populations are completely susceptible to the available acaricides or at least to two or three chemical classes (Centenaro et al., 2022). This leads to the following question: how feasible is it to apply a strategic tick control protocol on a farm in which ticks are resistant to six classes of acaricides? (Reck et al., 2014a). The application of strategic control protocols against multiple acaricide-resistant cattle ticks may require special attention regarding the expectations of farmers and practitioners. In these cases, the use of drugs with less than 100% efficacy would be necessary, and IPM practices must play a key role on the farm (Centenaro et al., 2022; Andreotti et al., 2024).

The classical concept of strategic tick control refers to the planning of tick treatments rather than arbitrary treatment. This is generally based on the prioritization of the treatments in the seasons ecologically unfavorable to the ticks (e.g., winter or dry season) (Nava et al., 2020; Nava et al., 2021; Centenaro et al., 2022). At least in theory, the combination of these principles would reduce the number of acaricide treatments and increase the efficacy of tick control. Nevertheless, there is a scarcity of data regarding the effects of tick control protocols on selective pressure for acaricide resistance. Currently, the rotation of drugs from different chemical classes is indicated to delay the drug resistance, although little data supports the effect of it on tick resistance (Thullner et al., 2007; Jonsson et al., 2010; Centenaro et al., 2022).

In this context, we need to revisit strategies of tick control under field conditions and validate novel protocols to provide technical information for those working in the field. This has particular importance in a world where multiple resistance to several classes of acaricides is a reality for a significant proportion of farms (Klafke et al., 2017). Noteworthy, these updated protocols must have two inseparable aims: reduce the tick burden while minimizing the selection of resistant tick populations.

## Integrated Parasite Management for *R. (B.) microplus* Control

The development of multiple resistance in tick populations can pose significant challenges for tick management in animal husbandry. To address this issue, IPM strategies are often recommended (Reck et al., 2014b; Rodriguez-Vivas et al., 2018; Centenaro et al., 2022). These strategies involve a combination of procedures, including rotating different classes of acaricides, using non-chemical control methods, and monitoring for resistance development.

Common strategies that can be applied as methods for cattle tick control other than the use of synthetic acaricides include pasture management (rotating cattle among paddocks to break the life cycle of ticks) (Andreotti et al., 2024), increasing host resistance (selecting cattle breeds or individuals with natural resistance or tolerance to ticks) (Cardoso et al., 2021), biological control (introducing natural enemies of ticks, such as beneficial fungi and or nematodes, and maintaining suitable habitat for insectivorous birds and other tick predators) (Webster et al., 2015; Camargo et al., 2016; Marciano et al., 2021; Filgueiras et al., 2023; Barbieri et al., 2023), the use of natural products (especially essential oils and compounds found in essential oils) (Klafke et al., 2021; Gonzaga et al., 2023), vaccination (Parizi et al., 2012; Arocho Rosario et al., 2022), education and training (training the farm’s personnel in proper tick identification, monitoring techniques, and the correct application of acaricides, and informing them about the best practices in tick control).

Among the methods listed above, the use of entomopathogenic fungi and the development of a vaccine for tick control seem to be the most promising alternative methods. Despite this, the main challenges for vaccines are identifying suitable antigens and reaching a high efficacy level. The main challenges for the biological control of ticks are the slow speed of kill, the need for highly concentrated agents, and specific legislation for registration. Currently, there is no registered product based on entomopathogenic fungi specifically for tick control in the country. According to the Brazilian Ministry of Agriculture, there are more than 190 registered products with entomopathogenic fungi (*Metarhizium* spp., *Beauveria* spp., and *Isaria/Cordyceps* spp.) (Brasil, 2023b) for controlling insect pests in agriculture, and none of them is registered exclusively for tick control.

Regarding botanical compounds, there are registered acaricides containing terpenes (citronellal and geraniol) associated with formulations with pyrethroids and organophosphates (Brasil, 2023a). There are also commercial formulations containing synthetic pyrethroids and organophosphates combined with piperonyl butoxide (Brasil, 2023a), which is a synergistic molecule synthesized from the phenylpropanoid safrole (Gonzaga et al., 2023).

Whilst these methods may not individually provide an elevated level of control, their integration into the cattle tick management program enables farmers to effectively handle resistant tick infestations (Webster et al., 2015). This approach not only mitigates the hazards linked to an excessive dependence on synthetic acaricides but also diminishes the likelihood of acaricide resistance development in tick populations. By incorporating diverse strategies, farmers can achieve a balanced and sustainable approach to cattle tick control, promoting both effectiveness and long-term resilience in pest management practices.

## Future Perspectives and Conclusion

**Figure 3:**
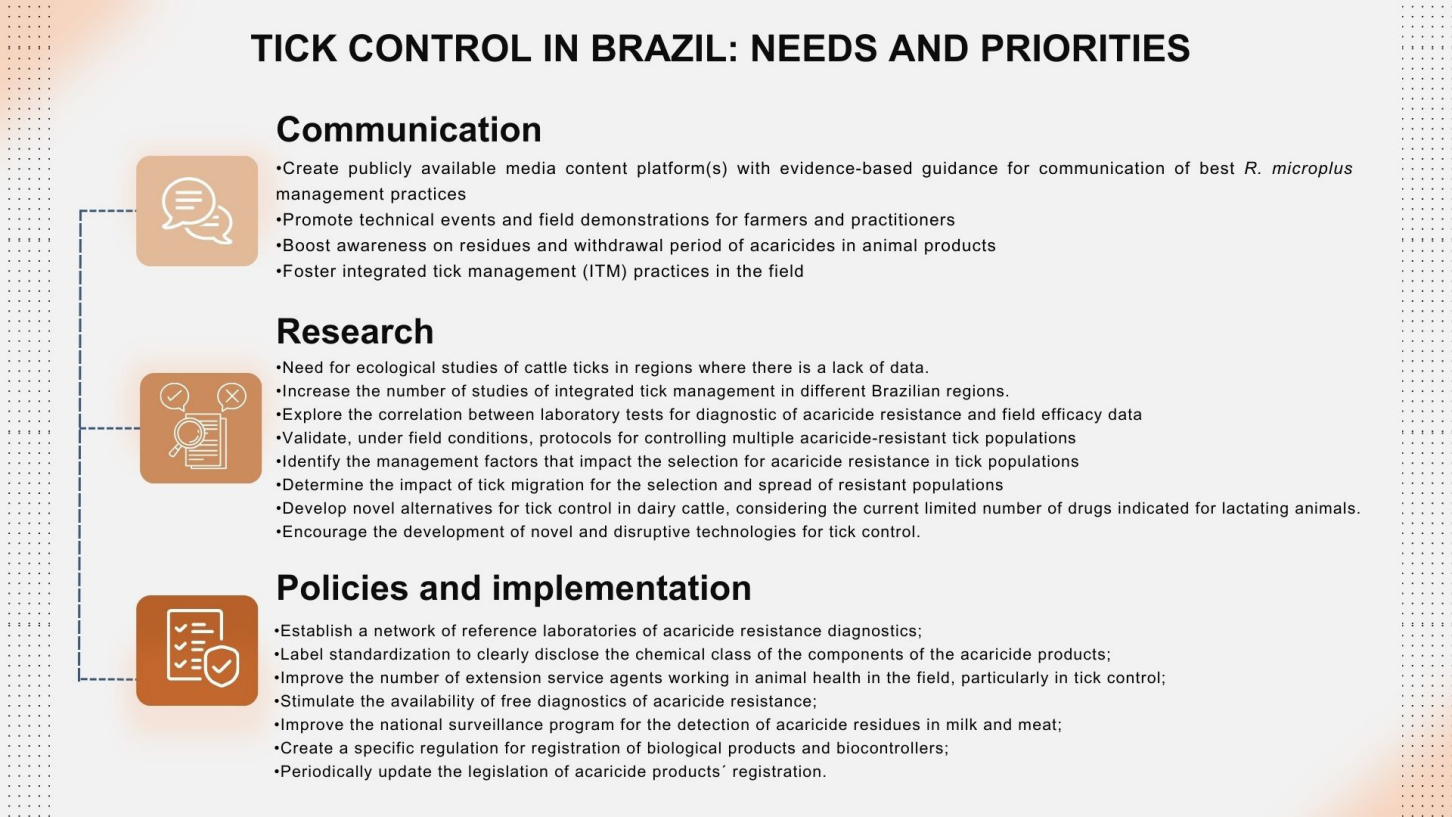
Tick control in Brazil: needs and priorities.

In conclusion, the challenges associated with cattle tick control in the field can be attributed to five key factors: (i) the prevalence of misinformation regarding tick control strategies, (ii) the insufficient adoption of IPM practices, (iii) the improper use of chemical acaricides, (iv) the low availability of technical support for farmers, and (iv) the emergence of multiple acaricide resistant tick populations. Addressing these issues is crucial for achieving an enhanced state of cattle tick control in Brazil, as illustrated in Figure 3. To this end, a comprehensive set of needs and priorities for effective cattle tick control can be delineated across three major domains: communication, research, and policies. By strategically addressing these aspects, we can pave the way for a more robust and sustainable approach to managing cattle tick infestations in Brazil.

## Supplementary Material

Supplementary material accompanies this paper.

Supplementary Table 1 History of acaricide resistance in cattle tick

Supplementary Table 2 Acaricide resistance status in Brazilian states

This material is available as part of the online article from https://doi.org/10.1590/S1984-29612024026
