# Supplementary material for: Brazil's battle against Rhipicephalus (Boophilus) microplus ticks: current strategies and future directions
Source: Rev Bras Parasitol Vet. 2024 Jun 21;33(2):e001423. doi: 10.1590/S1984-29612024026 (PMC11253825; doi:10.1590/S1984-29612024026)
Supplement: Supplementary Table 1 [file rbpv-33-2-e001423-Suppl.pdf]

Supplementary Tables

Supplementary Table 1. History of acaricide resistance in cattle tick

**Acaricide group**	**Market launch**	**1st report of resistance**[1]	
		**Brazil**[2]	**Other countries**[3]
Organophosphates	1950	1972 – Rio Grande do Sul (Arteche, 1972)	**1964** – Australia (Shaw & Malcolm, 1964)
Formamidines (amitraz)	1977	1993 – Rio de Janeiro	**1981** – Australia (Nolan, 1981)
Pyrethroids	1981	**1989** – Rio Grande do Sul	**1989** – Australia (Nolan, 1989)
Macrocyclic lactones	1980	**2001** – Rio Grande do Sul (Martins & Furlong, 2001)	2010 – Mexico (Perez-Cogollo et al., 2010)
Phenylpyrazoles (fipronil)	1996	**2004** – Minas Gerais	2007 – Uruguay (Cuore et al., 2007)
Benzoyl-phenylureas (fluazuron)	1994	**2014** – Rio Grande do Sul (Reck et al, 2014a)	2022 – Argentina (Torrents et al., 2022)

[1]Indexed scientific articles or freely available conference papers were considered in this history. The authors can provide the references for resistance reports upon request.

[2]Presented as “year – state (reference)”.

[3]Presented as “year – country (reference)”.

Supplementary Table 2. Acaricide resistance status in Brazilian states

Brazilian state	Chemical classes with report of tick resistance	References (methodology)
Acre	No information	-
Alagoas	SP; OP; Am	Silva et al., 2020 (AIT)
Amapá	No information	-
Amazonas	No information	-
Bahia	SP; OP; Am; FIP	Spagnol et al., 2010 (AIT); Raynal et al., 2013 (AIT)
Ceará	SP; OP; Am, ML	Higa et al., 2016 (AIT); Ferreira et al., 2022 (AIT)
Distrito Federal	SP, Am	Higa et al., 2016 (AIT)
Espírito Santo	SP; OP; Am	Higa et al., 2016 (AIT); Novato et al., 2022 (AIT)
Goiás	SP, Am, OP, Fip	Novato et al., 2022 (AIT); Nicarreta et al., 2023 (LPT and LIT)
Maranhão	SP, Am	Authors´ information
Mato Grosso	SP; OP; Am	Higa et al., 2016 (AIT)
Mato Grosso do Sul	SP; OP; Am; Fip; ML; Flu	Valsoni et al., 2021 (AIT, LIT)
Minas Gerais	SP; OP; Am; Fip; ML; Flu	Lopes et al., 2014; Maciel et al., 2016; Gonzaga et al., 2022
Pará	SP; Am	Sousa et al., 2022 (AIT)
Paraíba	SP, OP, Am, Fip, ML	Vilela et al., 2020 (LPT, LIT)
Paraná	SP; OP; Am; ML; Fip	Lovis et al., 2013 (LPT); Cruz et al., 2015 (field); Dolenga et al., 2022
Pernambuco	SP; OP; Am	de Santana et al., 2013 (AIT)
Piauí	SP; OP; Am	Authors´ information
Rio de Janeiro	SP, Am, OP	Gonçalves et al., 2004; Novato et al., 2022
Rio Grande do Norte	SP; Am	Coelho et al., 2013 (AIT)
Rio Grande do Sul	SP; OP; Am; Fip; ML; Flu	Reck et al., 2014a (AIT, Field, LPT); Webster et al., 2015 (Field); Klafke et al., 2017 (AIT, LIT, LPT)
Rondônia	SP; OP; Am; ML	Brito et al., 2011 (LPT); Gasparotto et al., 2020 (AIT)
Roraima	SP; Am	Junges et al., 2013 (AIT)
Santa Catarina	SP, Am, OP	Veiga et al., 2012 (AIT)
São Paulo	SP; OP; Am; Fip; ML	Castro-Janer et al., 2010 (LIT); Mendes et al., 2011 (LPT); Klafke et al., 2012 (LIT)
Sergipe	SP, Am, OP	Oliveira & Azevedo, 1998 (AIT)
Tocantins	SP; Am	Vilela et al., 2016 (AIT)

AIT, adult immersion test. LIT, larval immersion test. LPT, larval packet test.
SP, synthetic pyrethroids. OP, organophosphates. Am, amitraz. Fip, fipronil. ML, macrocyclic lactones. Flu, fluazuron.
